# Re-evaluation of IL-10 signaling reveals novel insights on the contribution of the intracellular domain of the IL-10R2 chain

**DOI:** 10.1371/journal.pone.0186317

**Published:** 2017-10-10

**Authors:** Ruud H. P. Wilbers, Debbie R. van Raaij, Lotte B. Westerhof, Jaap Bakker, Geert Smant, Arjen Schots

**Affiliations:** Wageningen University and Research, Plant Sciences Group, Laboratory of Nematology, Wageningen, The Netherlands; University of Colorado Denver School of Medicine, UNITED STATES

## Abstract

Interleukin-10 (IL-10) is an anti-inflammatory cytokine that plays a key role in maintaining immune homeostasis. IL-10-mediated responses are triggered upon binding to a heterodimeric receptor complex consisting of IL-10 receptor (IL-10R)1 and IL-10R2. Engagement of the IL-10R complex activates the intracellular kinases Jak1 and Tyk2, but the exact roles of IL-10R2 and IL-10R2-associated signaling via Tyk2 remain unclear. To elucidate the contribution of IL-10R2 and its signaling to IL-10 activity, we re-evaluated IL-10-mediated responses on bone marrow-derived dendritic cells, macrophages and mast cells. By using bone marrow from IL-10R^-/-^ mice it was revealed that IL-10-mediated responses depend on both IL-10R1 and IL-10R2 in all three cell types. On the contrary, bone marrow-derived cells from Tyk2^-/-^ mice showed similar responses to IL-10 as wild-type cells, indicating that signaling via this IL-10R2-associated kinase only plays a limited role. Tyk2 was shown to control the amplitude of STAT3 activation and the up-regulation of downstream SOCS3 expression. SOCS3 up-regulation was found to be cell-type dependent and correlated with the lack of early suppression of LPS-induced TNF-α in dendritic cells. Further investigation of the IL-10R complex revealed that both the extracellular and intracellular domains of IL-10R2 influence the conformation of IL-10R1 and that both domains were required for transducing IL-10 signals. This observation highlights a novel role for the intracellular domain of IL-10R2 in the molecular mechanisms of IL-10R activation.

## Introduction

Interleukin (IL)-10 is an essential regulator of the immune system, notably because of its anti-inflammatory properties and its role in re-establishing immune homeostasis. IL-10 is a strong suppressor of antigen presenting cells and lymphocytes [1, 2] and it was revealed that IL-10-deficient mice develop spontaneous inflammation in the intestine [3]. Besides its anti-inflammatory properties, IL-10 is also able to regulate proliferation of B cells, mast cells and NK cells [2, 4]. IL-10 signals through a heterodimeric receptor complex composed of IL-10 receptor (IL-10R)1 and IL-10R2 [5, 6]. Mice lacking either one of these two receptors develop spontaneous intestinal inflammation, alike IL-10-deficient mice [7, 8], which reveals a key role for IL-10 in controlling inflammatory diseases.

Engagement of the IL-10 receptor complex activates the Janus kinases Jak1 and Tyk2 [9, 10], which are associated with IL-10R1 and IL-10R2, respectively [11]. IL-10’s anti-inflammatory properties were shown to be dependent on the activation of Jak1 and the transcription factor STAT3 as macrophages deficient in STAT3 or JAK1 are unresponsive to IL-10 [12]. A role for the IL-10R2-associated kinase Tyk2 is more elusive. Karaghiosoff and co-workers showed that Tyk2-deficient mice develop normally and that the ability of IL-10 to suppress LPS-induced TNF-α expression in macrophages is not impaired [13]. However, Shaw and co-workers showed that IL-10 was not able to suppress nitric oxide production upon stimulation with a high dose of IFN-γ in macrophages lacking Tyk2 [14]. Therefore, the exact contribution of IL-10R2 or its signaling via Tyk2 in IL-10-mediated responses remains unclear.

The biological activity of IL-10 can be investigated in a variety of assays, but most common assays use mast cell or macrophage cell lines. The mast cell line MC/9 is routinely used to study the induction of proliferation by IL-10 [4, 15], whereas various macrophage cell lines are used to study IL-10’s anti-inflammatory properties [16, 17]. In some cases cell lines are transfected with plasmids for the expression of the native IL-10R's or using chimeric constructs that employ the intracellular domain of interferon-γ receptors instead of IL-10R's [6, 15, 18]. One might question the appropriateness of the use of cell lines in research on the mechanisms of cellular responses of IL-10. It is doubtful whether cell lines respond similar to *in vivo* cells as many cell lines are already cultured for a long time in different labs under different culturing conditions. The only selection pressure that these cells have encountered is efficient growth, and in the meantime these cell lines might have acquired (epi)genetic changes [19]. Cell lines could therefore have lost the ability to respond alike their *in vivo* counterparts.

Previously, we have reported that a stable monomeric form of human IL-10 (IL-10m) lacks the ability to suppress LPS-induced TNF-α in a macrophage cell line, whereas dimerization of this monomer via fusion to the Fc portion of IgA restored its activity [16]. In contrast, stable monomeric IL-10 was reported to have activity on a B cell line that was either stably transfected with human or murine IL-10R1 [18]. This observation raised the question whether these overexpressing cell lines are indeed a reliable model system to investigate IL-10-mediated responses. Re-evaluation of IL-10 activity on *ex vivo* cells could therefore give new insights on the molecular mechanisms of IL-10-mediated responses.

To re-evaluate IL-10-mediated responses and to particularly investigate the role of IL-10R2 we set-up biological activity assays for IL-10 using *ex vivo* cells. We differentiated mast cells, macrophages and dendritic cells from bone marrow and investigated their response to IL-10. As expected, IL-10 activity depends on both IL-10R1 and IL-10R2, but the IL-10R2-associated Tyk2 kinase only played a limited role in IL-10-mediated responses. However, we do show that Tyk2 contributes to early IL-10-mediated responses. Further investigation of the IL-10 signaling complex revealed that interactions between IL-10R1 and IL-10R2 (both intracellular and extracellular) reduce cellular binding of IL-10 as well as the binding of a monoclonal antibody against IL-10R1. IL-10R2 could therefore mediate conformational changes of the extracellular domain of IL-10R1, which are necessary to initiate IL-10 signaling.

## Material and methods

### Mice

Wild-type C57BL/6J mice were bred and maintained under specific pathogen-free conditions in the animal facilities at Wageningen University. IL-10R1^-/-^ mice were kindly provided by dr. W. Muller (University of Manchester), IL-10R2^-/-^ mice were obtained from Genentech (San Francisco, CA, USA) and bones from TYK2^-/-^ and STAT1^-/-^ mice were kindly provided by dr. B. Strobl (University of Veterinary Medicine Vienna). All experiments were approved by and conducted in accordance with relevant guidelines and regulations of the institutional animal care body at Wageningen University. The experiments of this specific study were approved by the animal experiments committee (DEC) of Wageningen University & Research.

### Cell culture

Mice were sacrificed by cervical dislocation after which bone marrow was isolated from the femur and tibia of 6–12 week old mice. Bone marrow derived macrophages (BMMΦ’s) were differentiated at 37°C/5% CO_2_ in RPMI-1640 medium containing 4 mM L-glutamine, 25 mM HEPES and supplemented with 10% fetal calf serum, 50 μM β-mercaptoethanol, 50 U/ml penicillin and 50 μg/ml streptomycin and 20% spent medium from L929 cells (ATCC). Bone marrow cells were seeded at 1×10^6^ cells/ml in 6- or 96-well tissue culture plates and cultured for 6 days, while refreshing medium at day 3. After 6 days of culture ≥95% of the cells expressed the macrophage markers F4/80 and CD11b.

Bone marrow derived dendritic cells (BMDC's) were differentiated for 10 days as described [20] using 10% spent medium from murine GM-CSF transfected X63 cells [21]. X63-GM-CSF cells were kindly provided by dr. M. Lutz (University of Erlangen-Nuremberg) with approval of dr. B. Stockinger (MRC National Institute for Medical Research). Briefly, bone marrow cells were plated at 2×10^5^ cells/ml in bacteriological petri dishes and incubated at 37°C/5% CO_2_. At day 3, 6 and 8 medium was refreshed and at day 10 both adherent and non-adherent cells were harvested. At this time typically ~90% of the cells expressed the dendritic cell markers CD11c and MHC class II.

Bone marrow-derived mast cells (BMMC) were differentiated at 37°C/5% CO_2_ using 1 ng/ml IL-3 (R&D Systems). Bone marrow cells were plated at 4×10^5^ cells/ml in bacteriological petri dishes and non-adherent cells were sub-cultured for at least 5 weeks, while refreshing growth medium twice a week. After 5 weeks of culture ≥95% of the cells expressed the mast cell markers FcεRI and c-kit.

Chinese hamster ovary cells (CHO-K1; obtained from DSMZ) were maintained at 37°C with 5% CO_2_ in RPMI-1640 medium containing 4 mM L-glutamine, 25 mM HEPES and supplemented with 10% fetal calf serum, 50 μM β-mercaptoethanol, 50 U/ml penicillin and 50 μg/ml streptomycin. Cells were sub-cultured three times a week by harvesting with trypsin.

### Expression and purification of monomeric IL-10

The sequence encoding the mature protein of stable monomeric human IL-10 was digested from pHYG-hFcα-IL-10m [16] and was cloned in frame with the *Arabidopsis thaliana* chitinase signal peptide (cSP) followed by a 6x histidine and FLAG tag as previously reported for IL-22 [22]. The construct was used to express IL-10m in 5–6 week old *Nicotiana benthamiana* plants by means of agroinfiltration. Plant produced IL-10m was extracted from the leaf apoplast (intercellular space) and purified using Ni-NTA Sepharose (IBA Life Sciences) as reported for IL-22 [22].

### LPS stimulation assays

BMMΦ’s were differentiated in 96 well plates and BMDC’s were seeded in 96 well plates at a density of 5x10^4^/well. Cells were pre-treated for 15 min. with recombinant IL-10 (R&D Systems) and subsequently stimulated with 100 ng/ml lipopolysaccharide (LPS from *E*. *coli* K12; Invivogen). After 2 hours or overnight stimulation, supernatants were analysed for TNF-α using the Ready-Set-Go!® ELISA kit (eBioscience) according to the supplier's protocol.

### Mast cell viability assay

BMMC’s were seeded in 96 well plates at a density of 1.5×10^5^ cells/well. Mast cells were then cultured in the presence of IL-10 or 1 ng/ml IL-3. After 48 hours cell viability was assessed by using the CellTiter 96® aqueous one solution (Promega) according to the supplier's protocol.

### STAT activation

Bone marrow-derived cells were treated for 20 min. with IL-10 (0, 1, 10 or 100 ng/ml). Cells were lysed using 1x Cell Lysis Buffer (Cell Signaling Technology) and total soluble protein content in the lysates was determined by the BCA method (Pierce). Proteins were separated on 12% Bis-Tris gels followed by transfer to a PVDF membrane by a wet blotting procedure. Thereafter the membrane was blocked in PBS (containing 0.1% v/v Tween-20 and 5% w/v non-fat dry milk powder) for 1 hour at room temperature, followed by overnight incubation at 4°C with monoclonal antibodies specific for STAT1, phospho-STAT1^Y701^, STAT3, phospho-STAT3^Y705^, STAT5 or phospho-STAT5^Y694^ in PBS (containing 0.1% v/v Tween-20 and 1% w/v BSA). All STAT antibodies were obtained from Cell Signaling Technology. A HRP-conjugated donkey anti-rabbit IgG (Jackson ImmunoResearch) was used as a secondary antibody. Blots were visualised in the G:BOX Chemi System (Syngene) using the SuperSignal West Femto substrate (Pierce).

### Construction of IL-10R expression vectors and transfections

The open reading frame of mouse IL-10R1, IL-10R2, IL-10R2^Δ230–330^ and IL-10R2^Δ1–190^ were amplified from mouse spleen cDNA using the Expand High Fidelity PCR System (Roche). The IL-10R2^Δ1–190^ fragment was cloned in frame with the native signal peptide by means of overlap extension PCR. Sense and antisense primers included BamHI and NheI restriction sequences respectively for subsequent cloning into the pMAX expression vector (Lonza). CHO-K1 cells were co-transfected with 0.5 μg/plasmid using the jetPRIME® transfection reagent (PolyPlus Transfection) using the supplier's protocol. 24 hours after transfection, CHO-K1 cells were analysed for IL-10R expression by flow cytometry.

### Flow cytometry

Bone marrow derived cells were stained in FACS buffer (PBS containing 0.1% BSA and 5mM EDTA) using the following monoclonal antibodies for cell surface markers (all obtained from eBioscience): PE-conjugated anti-CD11b, APC-conjugated anti-F4/80, PE-conjugated anti-CD11c, APC-conjugated MHC-II, PE-conjugated anti-FcεRI and APC-conjugated c-kit. Cells were first incubated with Fc receptor block (eBioscience) for 10 min to block any non-specific binding and subsequent staining steps were performed for 20 min. at 4°C, followed by washing with FACS buffer. An overview of the flow cytometric analysis of bone marrow-derived cells from all transgenic mice used in this study can be found in S3 Fig.

Upon CHO-K1 transfection, goat polyclonal anti-IL-10R1 and anti-IL-10R2 antibodies (R&D Systems) were used for detection of IL-10R expression followed by an APC-conjugated donkey anti-goat IgG secondary antibody (R&D Systems). Surface expression of IL-10R1 was also analysed by staining with a PE-conjugated monoclonal anti-IL-10R1 (1B1.3a; BioLegend). Intracellular staining of IL-10R2 was performed on methanol-permeabilized cells using a mouse monoclonal antibody that binds the cytoplasmic domain of IL-10R2 (clone F-6; Santa Cruz Biotechnology), followed by a PE-conjugated goat anti-mouse IgG secondary antibody (R&D Systems).

Surface labeling with IL-10 was performed by incubating 1.0 x10^6^ cells with 40 ng of IL-10 for 1 hour at 4°C. Surface-bound IL-10 was then cross-linked for 2 hours at 4°C with 2.5 mM BS3 (Pierce). Surface IL-10 was then detected with a goat polyclonal anti-IL-10 antibody (R&D Systems) followed by a PE-conjugated donkey anti-goat IgG secondary antibody (Jackson ImmunoResearch). CHO-K1 cells without IL-10 cross-linked to their surface were used as a negative control and mean fluorescent intensity obtained for these cells were subtracted from IL-10 labelled cells.

Stained cells were acquired using a Cyan-ADP Analyzer (Beckman Coulter) and analysed with FlowJo software (Tree Star, Inc.).

### Quantitative PCR

BMMΦ’s were differentiated in 6 well plates and 3x10^6^ BMDC’s were seeded in 6 well plates and were treated for 2 hours with IL-10 (0, 1, 10 or 100 ng/ml). Cells were then washed with PBS and mRNA was isolated with the RNeasy Mini kit (Qiagen) using the supplier’s protocol. A Turbo DNase I (Ambion) treatment was included to remove any residual DNA. Then cDNA was synthesized using the SuperScript III First-Strand Synthesis System (Invitrogen) according to the supplier’s protocol. Samples were analysed in triplicate for SOCS3 and HPRT (reference gene) expression by quantitative PCR using ABsolute SYBR Green Fluorescein mix (Thermo Scientific). Fold induction of SOCS3 expression was determined by the Pfaffl method [23].

### Data analysis

All data shown in the figures indicate the average of at least three biological replicates (*n*) that were determined by three technical replicates. In the figure legends *n* is indicated and error bars indicate the standard error. Significant differences between samples were calculated using the student’s t-test and regarded as significant when *P*<0.05. Significant differences are indicated in the figures by asterisks (*P*<0.05 (*) or *P*<0.01 (**)).

## Results

### Bone marrow-derived cells differentially respond to IL-10

**Fig 1:**
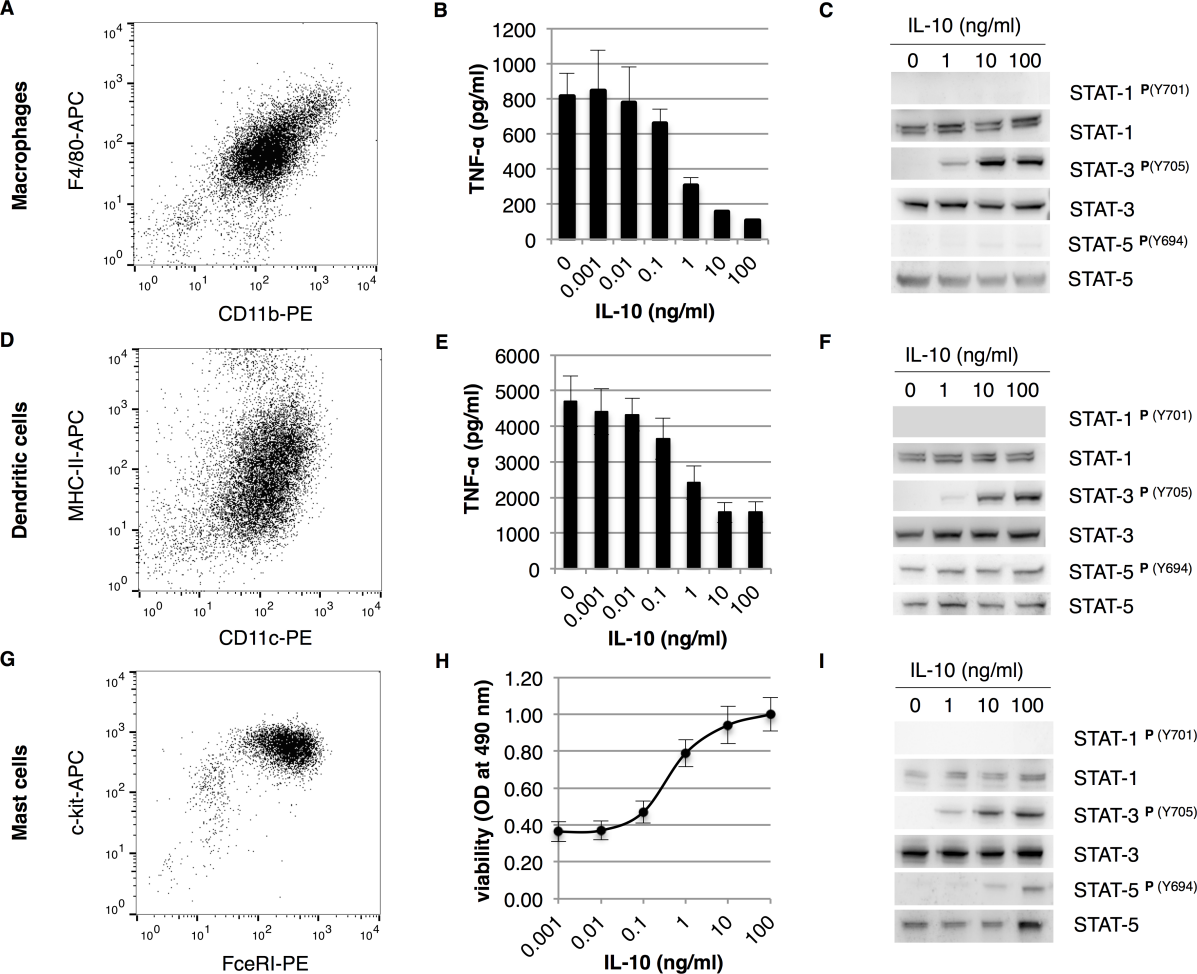
The differential response of bone marrow-derived cells to IL-10. Bone marrow-derived macrophages, dendritic cells and mast cells were analysed by flow cytometry for the expression of cellular markers CD11b & F4/80 (A), CD11c & MHC-II (D) and FcεRI & c-kit (G), respectively. Bone marrow-derived cells were tested for their response to IL-10 by measuring suppression of TNF-α expression by macrophages (B) and dendritic cells (E) or proliferative/anti-apoptotic ability in mast cells (H) (*n* = 4, error bars indicate standard error). Dose dependent tyrosine phosphorylation of STAT transcription factors by IL-10 (0, 1, 10 or 100 ng/ml) was analysed by western blot in macrophages (C), dendritic cells (F) and mast cells (I).

To set up biological activity assays for IL-10 based on *ex vivo* cells as an alternative for commonly used cell lines, mouse bone marrow-derived cells were differentiated into different cell types. To investigate the anti-inflammatory properties of IL-10, as commonly done on macrophage cell lines, bone marrow cells were differentiated into macrophages and dendritic cells. Flow cytometric analysis shows that ≥95% pure CD11b^+^ F4/80^+^ macrophages and ≥90% CD11c^+^ MHC-II^+^ dendritic cells were obtained (Fig 1A and 1D, respectively). Macrophages and dendritic cells were pre-treated with IL-10 and subsequently challenged with lipopolysaccharide (LPS). IL-10 inhibits TNF-α expression in a dose-dependent manner in both macrophages and dendritic cells (Fig 1B and 1E, respectively). We also observed that dendritic cells produced significantly more TNF-α when compared to macrophages, which is in line with the findings of Fleetwood and co-workers [24]. Furthermore, our data show that the maximum percentage inhibition of TNF-α levels is higher in macrophages than in dendritic cells (80% versus 60% in macrophages and dendritic cells, respectively). This is also comparable to the maximum of 60–70% suppression of TNF-α levels in human and mouse macrophages as previously published [16]. Next, we analysed the activation of STAT transcription factors by IL-10 in macrophages and dendritic cells. As expected, we observed strong tyrosine phosphorylation of STAT3^Y705^ in both cell types (Fig 1C and 1F). However, in contrast with existing literature we did not detect activation of STAT1 [14]. On the other hand, STAT5 was constitutively activated in dendritic cells, but no activation of STAT5 was observed in macrophages. This is likely because of the signaling cascade induced by GM-CSF [25], which is used to differentiate DC's from bone marrow.

To investigate proliferative responses of IL-10, which is typically done in MC/9 cells or B cell lines, bone marrow cells were differentiated into mast cells. Flow cytometric analysis showed that ≥95% pure c-kit^+^ FcεRI^+^ mast cells were obtained after 4 weeks of culture (Fig 1H). To test proliferative effects of IL-10, mast cells were cultured for 48 hours in the presence of IL-10 only, where after cell viability was assessed. IL-10 maintained viability of mast cells in a dose-dependent manner (Fig 1I). We also observed that the amount of viable cells was lower when compared to an IL-3 control (data not shown). This could indicate that IL-10 by itself does not induce proliferation, but only induces a survival signal in bone marrow-derived mast cells. When analysing IL-10 signaling in mast cells, we again observed strong activation of STAT3 and to a reduced extent of STAT5 (Fig 1I, only higher doses of IL-10). STAT1 was not activated in mast cells as was also observed in macrophages and dendritic cells. Further investigation of STAT1 involvement using bone marrow-derived cells from STAT1^-/-^ mice revealed that STAT1 is not required for IL-10-mediated responses on bone marrow-derived cells (S1 Fig). Altogether, these activity assays provide a solid basis for studying IL-10-mediated responses on *ex vivo* cells.

### Stable monomeric human IL-10 has impaired activity on *ex vivo* cells

**Fig 2:**
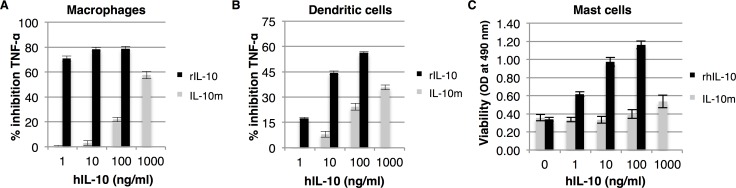
A stable monomeric form of human IL-10 has impaired activity. Bone marrow-derived macrophages, dendritic cells and mast cells from wild-type mice were tested for their response to human IL-10 and a stable monomeric form of IL-10 (IL-10m). Cells pre-treated with IL-10 were stimulated with 100 ng/ml LPS and TNF-α expression was determined to asses anti-inflammatory properties of IL-10 in macrophages (A) and dendritic cells (B) (*n* = 3, error bars indicate standard error). Mast cells were cultured for 48 hours in the presence of IL-10, where after cell viability was determined (C) (*n* = 3, error bars indicate standard error). *P*<0.01 at all concentrations in all three assays.

Stable monomeric IL-10 was previously reported to have activity on a B cell line that was either stably transfected with human or murine IL-10R1. Monomeric IL-10 had an EC_50_ value that was approximately 9 or 18 fold higher compared to the activity of recombinant IL-10 depending on which IL-10R1 was transfected into the cells [18]. To test the activity of a stable monomeric form of human IL-10 on *ex vivo* cells, we transiently expressed monomeric IL-10 in *Nicotiana benthamiana* and applied it to bone marrow-derived cell-based assays. These assays revealed that the activity of stable monomeric IL-10 on bone marrow-derived cells is strongly impaired. On mast cells, 1000-fold more IL-10m was required to obtain a similar response as 1 ng/ml recombinant human IL-10 from *E*. *coli* (Fig 2C). Similarly, when monomeric IL-10 was tested for anti-inflammatory responses on macrophages we only found strong suppression of TNF-α levels when we used up to 1 μg/ml monomeric IL-10 (Fig 2A). However, 1 ng/ml of recombinant human IL-10 was still significantly more potent in suppressing TNF-α expression compared to IL-10m (*P*<0.01). This is in in line with our previous observation that monomeric IL-10 is hardly able to suppress TNF-α expression in a murine macrophage cell line [16]. Furthermore, we also show that the anti-inflammatory properties of IL-10m were impaired in dendritic cells (Fig 2B), but the difference in activity with recombinant IL-10 is smaller. No differences in activity were observed between recombinant human and mouse IL-10 from *E*. *coli* (data not shown). Altogether we conclude that a stable monomeric form of IL-10 has impaired activity on *ex vivo* cells and that previously observed activity of this molecule might arise from differences in IL-10R expression levels.

### IL-10 requires both IL-10R's for activity, but not signaling via Tyk2

**Fig 3:**
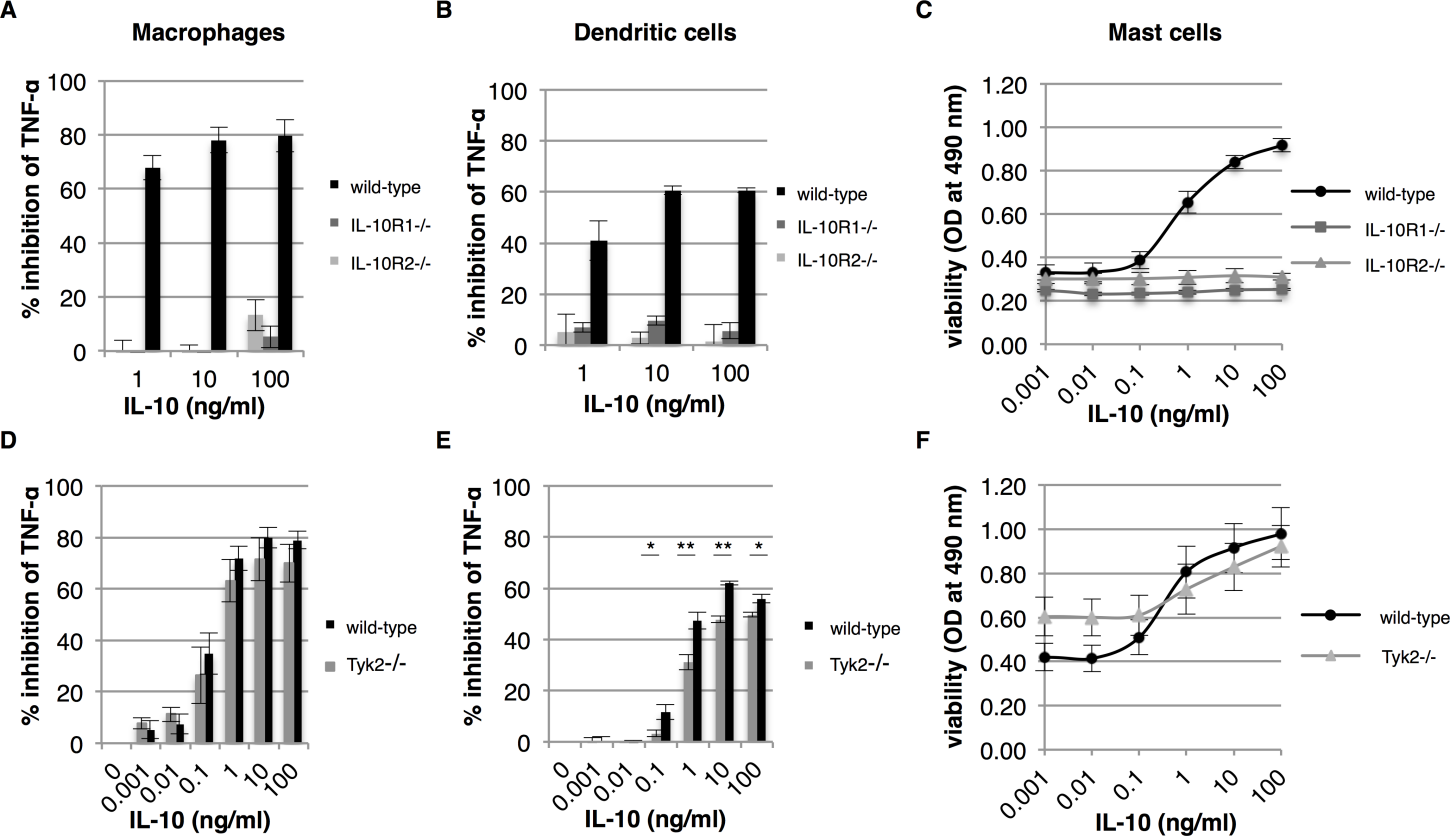
IL-10R2 mediated signaling via Tyk2 plays a limited role in IL-10 activity. Bone marrow-derived macrophages, dendritic cells and mast cells from wild-type, IL-10R1^-/-^, IL-10R2^-/-^ and Tyk2^-/-^ mice were tested for their response to IL-10. Macrophages and dendritic cells from wild-type and IL-10R^-/-^ mice were pre-treated with IL-10 and subsequently stimulated with 100 ng/ml LPS. The percentage of inhibition of TNF-α expression of macrophages and dendritic cells was determined after overnight incubation (A and B, respectively) (*n* = 3, error bars indicate standard error). Similarly, macrophages and dendritic cells from Tyk2^-/-^ mice were tested for their response to IL-10 (D and E, respectively) (*n* = 4, error bars indicate standard error). Mast cells from wild-type and transgenic mice were cultured for 48 hours in the presence of IL-10 and cell viability was determined (C and F) (*n* = 3 for IL-10R^-/-^ mice and *n* = 4 for Tyk2^-/-^ mice, error bars indicate standard error). Asterisk(s) indicate significant differences as determined by a Welch’s *t*-test (*P<0.05; **P<0.01).

To investigate the role of IL-10R's in IL-10-mediated responses on bone marrow-derived cells, we cultured macrophages, dendritic cells and mast cells from bone marrow of IL-10R1^-/-^ and IL-10R2^-/-^ mice and tested them for their response to IL-10. As expected, both IL-10R1 and IL-10R2 are required for IL-10’s anti-inflammatory responses in macrophages and dendritic cells (Fig 3A and 3B, respectively). In the absence of either one of the two IL-10 receptors, IL-10 is not able to suppress LPS induced TNF-α expression. Similarly, bone marrow-derived mast cells also depend on both IL-10R1 and IL-10R2 for activity of IL-10 (Fig 3C). As IL-10R2 is required for IL-10 activity, we further investigated whether IL-10R2 signaling via Tyk2 plays a role in our mast cell-based assay. Tyk2 is a member of the Janus kinase family of tyrosine kinases and to date is the only signaling molecule described to be associated with IL-10R2. We investigated the ability of IL-10 to maintain viability in mast cells in the absence of the Tyk2 kinase. Macrophages and dendritic cells were taken along, since literature is elusive on the role of Tyk2 in IL-10 anti-inflammatory responses. In the absence of IL-10R2-mediated signaling via Tyk2, IL-10 is perfectly capable to suppress TNF-α expression in macrophages and no significant differences were found between wild-type and Tyk2^-/-^ macrophages (Fig 3D). The percentage of inhibition of TNF-α after LPS stimulation is approximately 8% lower in Tyk2^-/-^ macrophages, but this is not significant. However, the percentages of inhibition of TNF-α of wild-type and Tyk2^-/-^ dendritic cells were significantly different. Although a similar dose-response curve was observed for both dendritic cells, the level of TNF-α inhibition in Tyk2^-/-^ dendritic cells was ~15% lower when compared to wild-type cells (Fig 3E). A similar reduction in IL-10-mediated responses was also observed in the mast cell based assay. At high concentrations of IL-10, Tyk2^-/-^ mast cells are slightly less viable than their wild-type counterparts, but this difference was not significant (Fig 3F). Altogether, we conclude that IL-10 requires both IL-10 receptors for activity, but there is only a minor role for the IL-10R2 associated Tyk2 kinase.

### Tyk2 controls the amplitude of early IL-10-mediated responses

**Fig 4:**
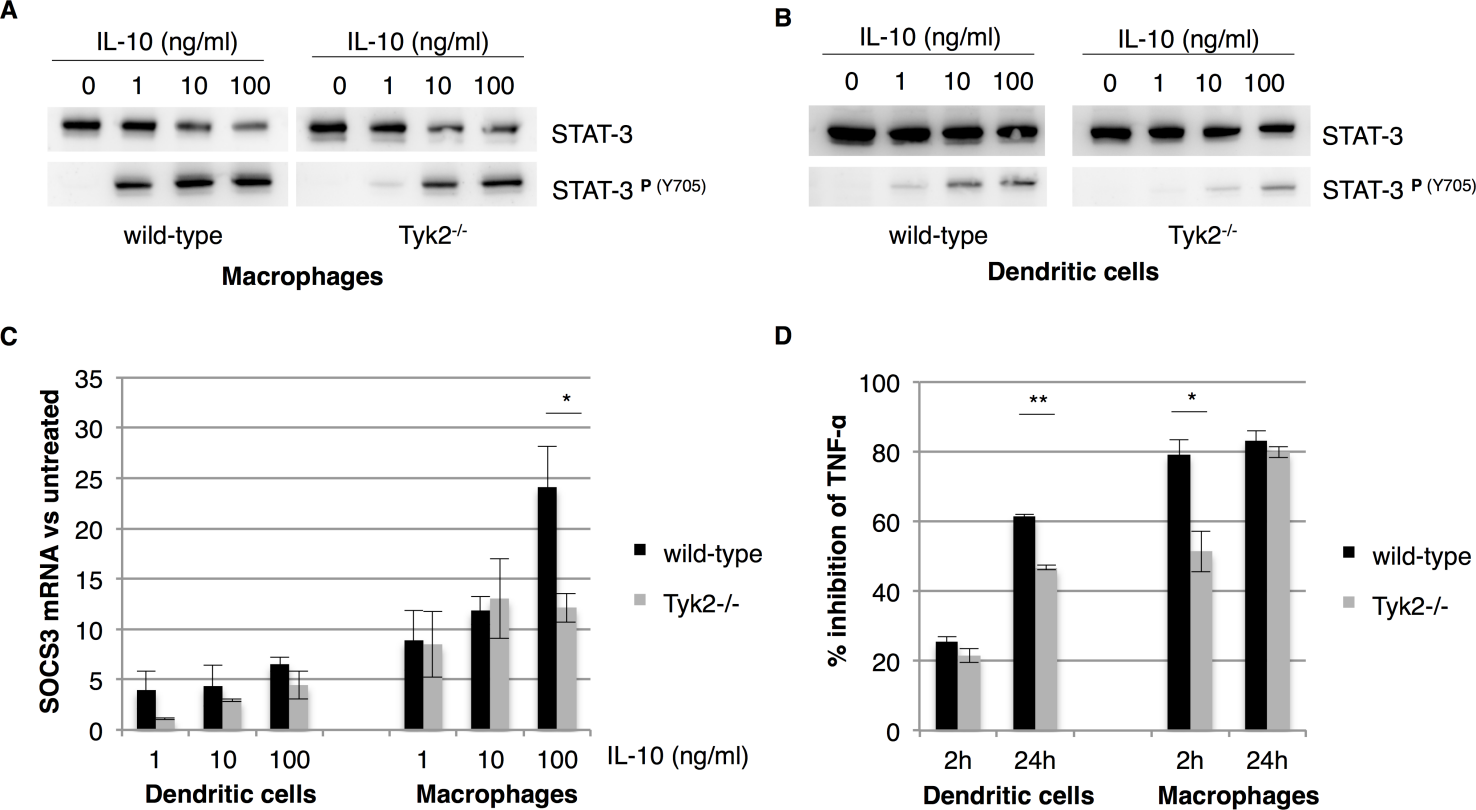
Tyk2 affects early responses to IL-10. Bone marrow-derived macrophages and dendritic cells from Tyk2^-/-^ mice were investigated in more detail for their signaling. Phosphorylation of tyrosine 705 (Y705) of STAT3 by IL-10 (0, 1, 10 and 100 ng/ml) was analysed in wild-type and Tyk2^-/-^ macrophages and dendritic cells by western blot (A and B respectively). Relative up-regulation of SOCS3 mRNA expression by IL-10 was analysed by quantitative PCR in both macrophages and dendritic cells (C). Fold induction of SOCS3 expression was calculated using the 2^ΔCt^ method using HPRT as a reference gene. Macrophages and dendritic cells from wild-type and Tyk2^-/-^ transgenic mice were pre-treated with IL-10 and were stimulated with 100 ng/ml LPS and the inhibition of TNF-α expression was determined at 2 and 24 hours (D). Asterisk(s) indicate significant differences as determined by a Welch’s *t*-test (*P<0.05; **P<0.01).

Deficiency of Tyk2 in bone marrow-derived cells only plays a limited role in IL-10-mediated responses and this is in line with a previous report by Karaghiosoff and co-workers [13]. They also showed that IL-10 is able to suppress LPS induced TNF-α expression in Tyk2^-/-^ macrophages. In contrast, Shaw and co-workers revealed that Tyk2^-/-^ macrophages are unable to suppress IFN-γ-induced nitric oxide synthesis at a high dose of IFN-γ [14]. We therefore investigated IL-10-mediated responses in Tyk2^-/-^ dendritic cells and macrophages in more detail to elucidate the contribution of Tyk2 to IL-10 activity. First, we determined the dose-dependent phosphorylation of the transcription factor STAT3 (Y705) in both wild-type and Tyk2^-/-^ macrophages and dendritic cells (Fig 4A and 4B, respectively). In both cell types we observed a ~10-fold reduction in STAT3 phosphorylation when cells are Tyk2 deficient. Furthermore, STAT3 phosphorylation in dendritic cells is rather poor when compared to macrophages, but IL-10 is perfectly capable to suppress TNF-α in those cells (even in the absence of Tyk2). We therefore conclude that Tyk2 is required for optimal IL-10-induced STAT3 tyrosine phosphorylation.

To investigate whether Tyk2 is required for optimal IL-10 induced gene expression we analysed the expression of suppressor of cytokine signaling 3 (SOCS3) expression. SOCS3 is strongly up-regulated by IL-10 and is one of the major factors that mediates IL-10’s anti-inflammatory functions [2]. In Fig 4C we show that SOCS3 is upregulated in a dose-dependent manner in both dendritic cells and macrophages and that Tyk2 deficiency significantly reduces the upregulation of SOCS3 in macrophages at a concentration of 100 ng/ml IL-10. Surprisingly, SOCS3 upregulation is significantly reduced in dendritic cells when compared to macrophages regardless of Tyk2 deficiency (*P*<0.05 at 10 and 100 ng/ml IL-10). No significant differences were observed for SOCS3 upregulation in wild-type versus Tyk2 deficient dendritic cells. Tyk2 therefore plays a role in regulating IL-10 induced gene expression, but its role is cell type dependent.

The role of SOCS3 in IL-10-mediated responses was previously investigated by Qasimi and co-workers (2006). In the case of TNF-α suppression SOCS3 seemed to play a role in early inhibition [17]. We therefore investigated whether Tyk2 deficiency would influence the suppression of TNF-α only 2 hours after LPS stimulation. In Fig 4D we show that Tyk2 is indeed required for the early inhibition of LPS-induced TNF-α in macrophages. At 2 hours after stimulation IL-10 already supresses TNF-α by 80% in wild-type macrophages, but in Tyk2^-/-^ macrophages this is significantly reduced to 50%. Furthermore, we show that IL-10 is unable to suppress TNF-α at an earlier time point in dendritic cells, which is in line with impaired SOCS3 upregulation in these cells. All together we conclude that Tyk2 optimizes STAT3 signaling and induction of SOCS3 expression and thereby controls the amplitude of one the main anti-inflammatory signaling cascades Jak1-STAT3-SOCS3 in macrophages.

### The extracellular domain of IL-10R2 is not sufficient to maintain IL-10 activity

**Fig 5:**
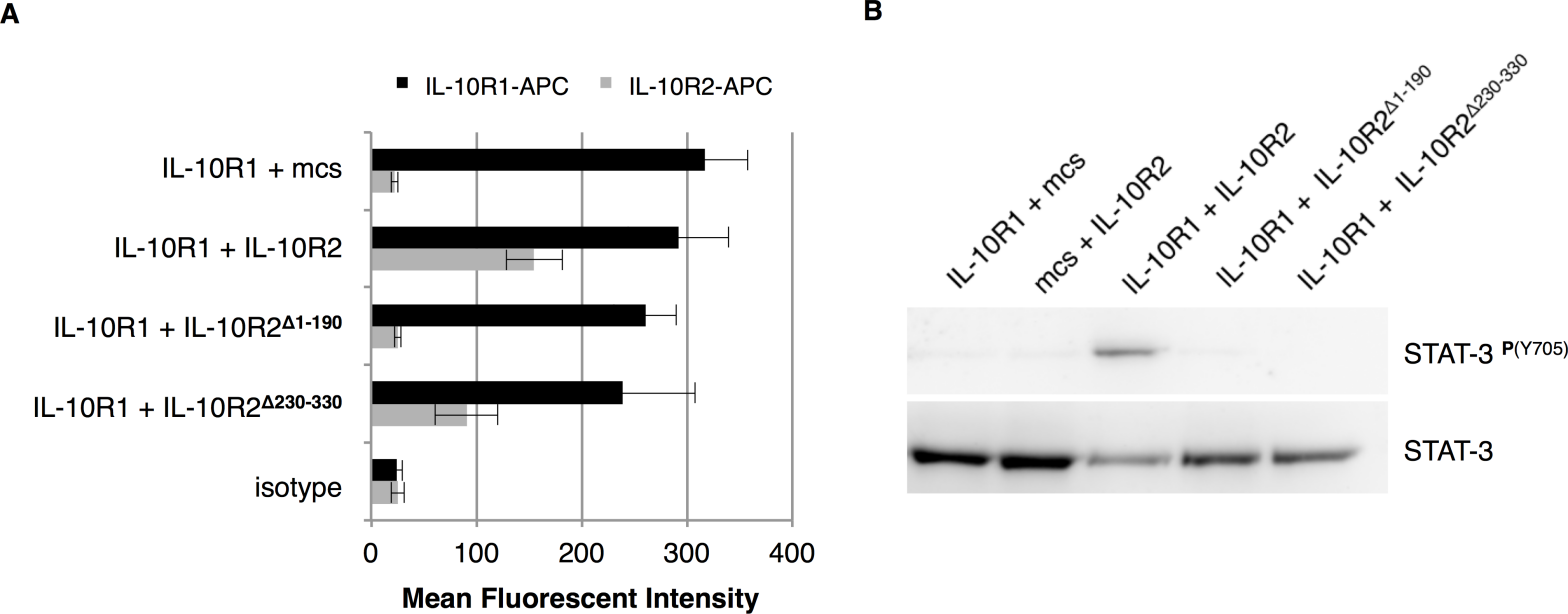
The extracellular domain of IL-10R2 is not sufficient to maintain IL-10 activity. CHO-K1 cells were co-transfected with combinations of the expression vectors for IL-10R1, IL-10R2, IL-10R2^Δ230–330^ (extracellular domain), IL-10R2^Δ1–190^ (intracellular domain) or an empty vector (mcs) and cultured for 24 hours. Surface expression of IL-10R1 or IL-10R2 was analysed by flow cytometry and mean fluorescent intensity is plotted (*n* = 6, error bars represent standard error) (A). Phosphorylation of tyrosine 705 (Y705) of STAT3 by IL-10 (100 ng/ml) in CHO-K1 cells upon transient transfection with IL-10 receptors was analysed by western blot (B).

As IL-10R2-mediated signaling via Tyk2 only plays a limited role in IL-10 mediated responses, we hypothesised that the intracellular domain of IL-10R2 may be dispensable for activity. Krause and co-workers reported that IL-10R1 and IL-10R2 pre-assemble in the cell membrane prior to the binding of IL-10 [26]. We hypothesized that the extracellular domain of IL-10R2 might be sufficient to form a signaling complex with the high affinity IL-10R1. To investigate whether IL-10-mediated signaling could be triggered from a receptor complex that lacks the intracellular domain of IL-10R2 we co-transfected CHO-K1 cells with expression vectors for IL-10R1 in combination with IL-10R2, IL-10R2^Δ230–330^ (extracellular domain), IL-10R2^Δ1–190^ (intracellular domain) or an empty vector control (mcs). Upon transfection, surface IL-10R expression was analysed by flow cytometry (Fig 5A). No significant differences were observed in surface IL-10R1 expression upon co-transfection with different IL-10R2 constructs. Furthermore, surface IL-10R2 expression was only detected for cells that were transfected with the complete or the IL-10R2^Δ230–330^ construct (lacking the intracellular domain). Cells were also stained simultaneously for IL-10R1 and IL-10R2 to check for the efficiency of co-transfection (S2A Fig). The cytoplasmic domain of IL-10R2 was stained intracellular to confirm expression of the IL-10R2^Δ1–190^ construct (S2B Fig). Next we treated transfected CHO-K1 cells with 100 ng/ml IL-10 to induce STAT3 phosphorylation. Fig 5B reveals that STAT3 was only phosphorylated by IL-10 in CHO-K1 cells when IL-10R1 was co-transfected with the full IL-10R2. We therefore conclude that both the extracellular and intracellular domains of IL-10R2 are required to initiate IL-10-mediated signaling. But as signaling can occur in the absence of the IL-10R2-associated kinase Tyk2, the exact role of the intracellular part of IL-10R2 remains to be elucidated.

### IL-10R2 mediates conformational changes of IL-10R1

**Fig 6:**
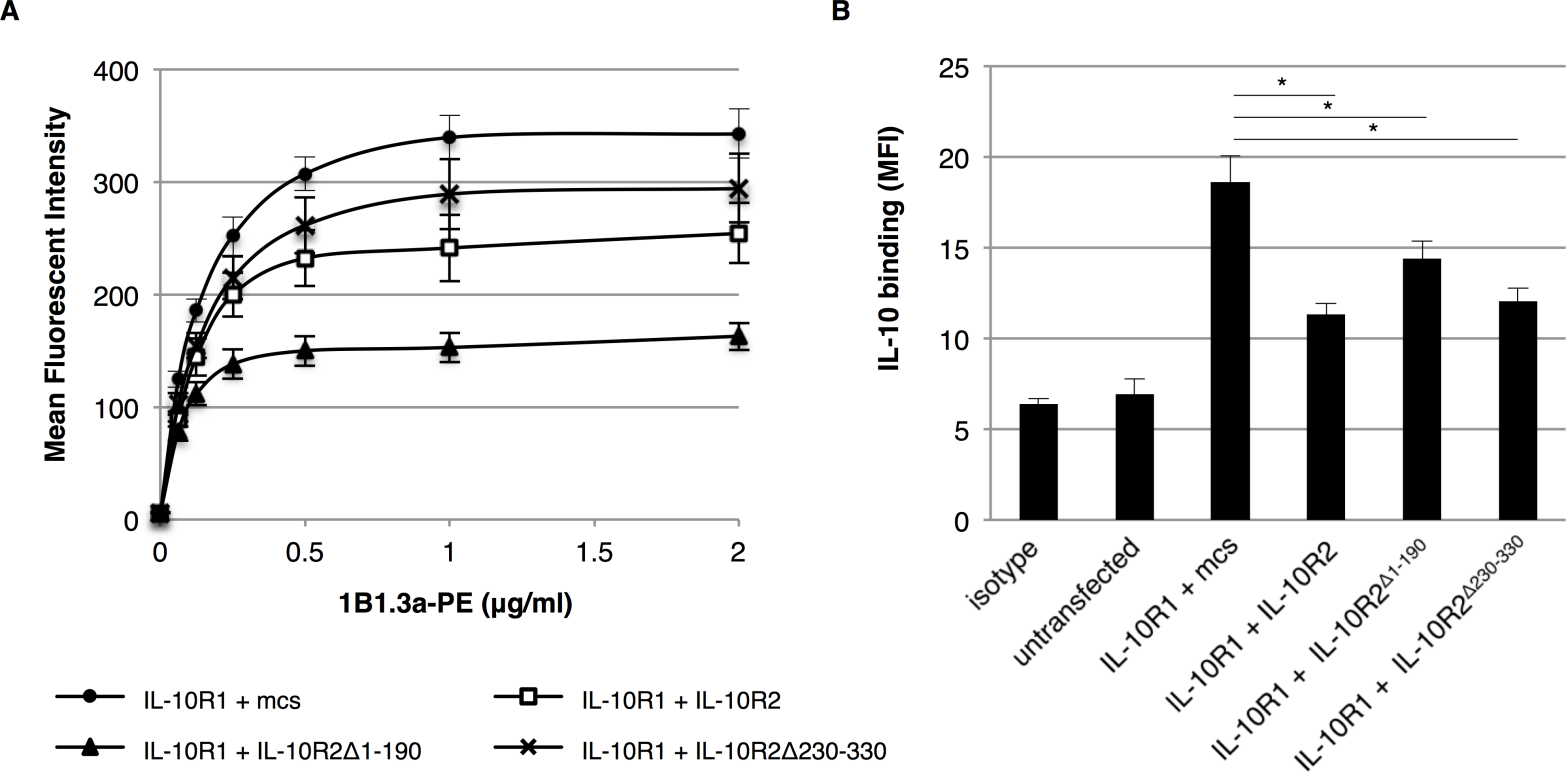
The intracellular domain of IL-10R2 mediates conformational changes in IL-10R1. CHO-K1 cells were co-transfected with combinations of the expression vectors for IL-10R1, IL-10R2, IL-10R2^Δ230–330^, IL-10R2^Δ1–190^ or an empty vector (mcs) and cultured for 24 hours. Cells were surface stained with the 1B1.3a anti-mouse IL-10R1 monoclonal antibody and analysed by flow cytometry (A). Mean fluorescent intensity for IL-10R1 binding is plotted in a dose-dependent manner (*n* = 6, error bars represent standard error). IL-10 was cross-linked to the surface of transfected cells and surface-bound IL-10 was detected by flow cytometry (B). Mean fluorescent intensity for IL-10 binding is plotted (*n* = 3, error bars represent standard error). Asterisk(s) indicate significant differences as determined by a Welch’s *t*-test (*P<0.05; **P<0.01).

A role for the intracellular domain of IL-10R2 may be found in the association with yet unidentified signaling components. In this manner, IL-10R2 could contribute to IL-10-mediated signaling independent of Tyk2. However, when staining transfected CHO-K1 cells with the monoclonal antibody 1B1.3a (binding IL-10R1) we made the striking observation that IL-10R1 detection was reduced by the co-expression of IL-10R2. We repeated the co-transfections and stained the cells with the 1B1.3a antibody (Fig 6A). Upon co-transfection of IL-10R1 and IL-10R2 we observed a significant reduction in maximum binding of the monoclonal 1B1.3a antibody (*P*<0.05 at all concentrations). As we show in Fig 5A, staining with a polyclonal anti-IL-10R1 antibody did not differ significantly between co-transfections, which indicates that there are no differences in overall expression of IL-10R1. The reduction in binding of the 1B1.3a antibody could be explained by steric hindrance of the extracellular domain of IL-10R2 or by conformational changes that are induced by IL-10R2. Yet, upon co-expression of IL-10R2 lacking an extracellular domain (IL-10R2^Δ1–190^) we observed an even stronger reduction of 1B1.3a binding (*P*<0.01 at all concentrations). We therefore believe that the intracellular domain of IL-10R2 mediates conformational changes of the extracellular domain of IL-10R1 that influence the binding of the 1B1.3a antibody to its epitope on IL-10R1.

To investigate whether these conformational changes mediated by the intracellular domain of IL-10R2 also influence the binding of IL-10 to its receptors, we labelled transfected CHO-K1 cells by cross-linking IL-10 onto the surface. Surface-bound IL-10 was then analysed by flow cytometry. Mean fluorescent intensity of surface-bound IL-10 is shown in Fig 6B. The maximum level of IL-10 binding was found for CHO-K1 cells transfected with only IL-10R1. Co-expression of all three IL-10R2 constructs significantly reduced the amount of surface-bound IL-10, but the reduction by the IL-10R2 lacking the extracellular domain (IL-10R2^Δ1–190^) was less prominent. In fact, the full IL-10R2 construct significantly reduces the amount of surface-bound IL-10 compared to IL-10R1 alone and upon co-transfection of the intracellular domain of IL-10R2. Altogether we conclude that interactions between IL-10R1 and IL-10R2, and most strikingly the intracellular domain of IL-10R2, mediate conformational changes of the extracellular domain of IL-10R1. These interactions seem to reduce the cellular binding of IL-10 to its receptors, but are required for transducing IL-10-mediated signaling.

## Discussion

Interleukin-10 (IL-10) plays a key role in maintaining immune homeostasis and mediates its activity by binding to a heterodimeric receptor complex consisting of IL-10R1 and IL-10R2. However, the exact contribution of IL-10R2, and in particular IL-10R2-associated signaling via Tyk2, to IL-10-mediated responses is still largely unclear. Therefore, we re-evaluated IL-10-mediated responses and investigated the contribution of IL-10R2. We developed cellular assays using mouse bone marrow-derived cells as commonly used cell lines might not be the most reliable model system. Our study shows that IL-10 is able to suppress LPS-induced TNF-α expression in bone marrow-derived macrophages and dendritic cells. However, subtle differences in the magnitude of TNF-α suppression were found between these two cell types. Next to IL-10’s anti-inflammatory properties, we show that IL-10 is also able to maintain viability in mast cells in the absence of IL-3. As expected, IL-10 induces strong activation of the transcription factor STAT3 in all three cell types, but IL-10 did not activate STAT1 or STAT5. The lack of STAT1 activation is in contrast to previous reports [13, 14], but the use of bone marrow-derived cells from STAT1^-/-^ mice in our study did not affect IL-10-mediated responses. We therefore conclude that STAT1 does not play a major role in the downstream signaling of IL-10.

Tyk2 signaling is investigated intensively and Tyk2-deficient mice mainly display impaired responses to interferons and IL-12 [13, 27]. However, the contribution of Tyk2 signaling to IL-10-mediated responses remains elusive. As previously reported by Karaghiosoff and co-workers IL-10 is not impaired in its ability to suppress LPS-induced TNF-α in macrophages from Tyk2^-/-^ mice [13]. Yet, when activated with high doses of IFN-γ, IL-10 is no longer able to suppress nitric oxide production in macrophages [14]. In our study we investigated the involvement of Tyk2 on three different cell types and our work demonstrates that Tyk2 only plays a limited role in IL-10-mediated responses. IL-10 activity on macrophages and mast cells was slightly reduced, but this was not found to be significant. Like in macrophages, we observed a 8–16% reduction in maximum suppression of LPS-induced TNF-α in dendritic cells, but for dendritic cells this difference was significant. Tyk2 does play a role in suppressing TNF-α expression in macrophages upon LPS stimulation, but only early IL-10 responses are affected. Qasimi and co-workers showed that early IL-10-mediated suppression of LPS-induced TNF-α is controlled by SOCS3 [17]. Indeed, IL-10 enhances SOCS3 expression in a STAT3 dependent manner [2, 28] and we observed a ~10-fold reduction in tyrosine phosphorylation of STAT3 (Y705) in Tyk2-deficient macrophages and dendritic cells. Furthermore, Tyk2-deficient macrophages also showed reduced up-regulation of SOCS3 expression, which in turn explains the altered early response to IL-10 in macrophages. In addition to a role in early suppression of TNF-α, SOCS3 also plays a role in IL-10-mediated suppression of nitric oxide production in LPS-stimulated macrophages [17]. Furthermore, SOCS3 is able to suppress IFN signaling [2, 29]. Impaired up-regulation of SOCS3 could therefore also explain why IL-10 is not able to suppress nitric oxide production in Tyk2-deficient macrophages upon stimulation with IFN-γ [14]. Altogether, we conclude that IL-10R2-associated signaling via Tyk2 controls the amplitude of one of the main anti-inflammatory signaling cascades Jak1-STAT3-SOCS3 in macrophages, which affects early responses of IL-10.

Previously we have reported that stable monomeric IL-10 is not able to suppress LPS-induced TNF-α expression in a macrophage cell line [16], but Josephson and co-workers showed that this monomer was able to induce proliferation in a B cell line transfected with either human or mouse IL-10R1 [18]. Monomeric IL-10 had an EC_50_ value that was approximately 9 or 18 fold higher compared to the activity of recombinant IL-10 depending on which IL-10R1 was used to transfect B cells. However, when we tested monomeric IL-10 on bone marrow-derived cells, we found that 100-1000-fold more monomeric IL-10 is required to obtain similar activity as recombinant IL-10. We cannot exclude that the N-terminal 6x histidine-FLAG tag of our monomeric IL-10 influences the activity, but we previously fused the Fc portion of human IgA to the N-terminus of IL-10 without affecting its activity [16]. IL-10R1 expression level has been reported to play a critical role in IL-10-mediated responses [15, 30]. For instance, neutrophils require enhanced IL-10R1 expression to respond to IL-10 [30]. The observed difference in activity for monomeric IL-10 in our study and the study of Josephson and co-workers likely arises from overexpression of IL-10R1 upon transfection in the latter study. Cellular assays based on *ex vivo* cells might therefore be more representative for the *in vivo* situation.

Monomeric IL-10 might be able to assemble the complete IL-10 receptor complex when IL-10R1 expression is high, but not under natural conditions when IL-10R expression is much lower. Crystallization studies of IL-10 with soluble (s)IL-10R1 revealed that a complex is formed consisting of 2 IL-10 dimers and 4 IL-10R1 chains [31]. On the contrary, monomeric IL-10 forms a complex with sIL-10R1 in a 1:1 ratio [18]. This would indicate that the signaling complex formed by monomeric IL-10 differs from the receptor complex with dimeric IL-10. We have previously shown that dimerization of monomeric IL-10 restores its ability to suppress LPS-induced TNF-α expression in macrophages [16]. This could indicate that dimerization of IL-10 is required for the assembly of a signaling complex that consists of multiple IL-10R subunits.

Complex assembly requires the initial binding of IL-10 to the high affinity receptor IL-10R1. This interaction mediates conformational changes within IL-10 that increase the binding affinity of IL-10R2 to the IL-10/IL-10R1 complex [32]. This structural information on IL-10 receptor complex assembly based on soluble receptors has been valuable, but this cannot reveal the rearrangement of receptors induced by cytokine binding. Furthermore, receptor rearrangements that take place inside the cell cannot be investigated in this way due to the lack of transmembrane regions and intracellular domains. Therefore, the exact mechanism by which IL-10 initiates the downstream activation of Jak1 and Tyk2 remains unknown. Our study now demonstrates that the conformation of the extracellular domain of IL-10R1 is influenced by interactions with IL-10R2 (both extracellular and intracellular). Co-expression of IL-10R2, or parts thereof, reduces the binding of IL-10 to IL-10R1 on the cell surface. The observation that the intracellular domain of IL-10R2 influences IL-10 binding to IL-10R1 might also explain why affinity of IL-10 towards IL-10R1 on intact cells is higher than for sIL-10R1 [33]. Although co-expression of both receptors results in reduced cellular binding of IL-10, the affinity for IL-10 to IL-10R1 on the surface might still be higher compared to the soluble extracellular domain of IL-10R1 used in crystallization studies. But above all, this observation highlights the importance of the intracellular domain in the molecular mechanism of IL-10R activation.

A common model for class I cytokine receptor activation has recently been proposed by Pang and co-workers [34]. This model of activation involves scissor-like rotation and self-rotation of the receptors. The intracellular domains of two receptor molecules are separated from each other by scissor-like rotation, allowing intracellular signaling components to bind. Self-rotation of the receptors arranges the Janus kinases in such an orientation that they can be *trans*-phosphorylated and initiates signaling. Although IL-10 belongs to class II cytokines, scissor-like rotation has been proposed as an activation mechanism [31]. However, Krause and co-workers demonstrated by confocal fluorescence spectroscopy that IL-10R1 and IL-10R2 are pre-assembled in the cellular membrane. Furthermore, they revealed that there are no major structural changes among the intracellular domains upon ligand binding [26]. It is therefore more likely that IL-10 receptors are activated by a different mechanism. In contrast, the receptor complex of IFN-γ undergoes major structural changes upon cytokine binding [35]. Binding of IFN-γ induces rearrangement of the intracellular domains of both IFN-γR1 and IFN-γR2 thereby allowing more space for downstream signaling components to bind. The rearrangement of intracellular receptor domains seems to differ between the signaling complexes of IL-10 and IFN-γ. We therefore reason that cell-based assays using hybrid IL-10 receptors harbouring the intracellular domains of their respective IFN-γR’s are not a reliable model to investigate IL-10 signaling.

In line with pre-assembly of IL-10R1 and IL-10R2 on the cellular surface, our study now reveals that co-expression of both receptor chains influences the conformation of the extracellular domain of IL-10R1. We also conclude that even though cellular binding of IL-10 is reduced by co-expression of IL-10R1 and IL-10R2, interactions between the extracellular and intracellular domains of both receptors are required to initiate signal transduction via Jak1 and Tyk2. Future investigation should now focus on how these interactions between IL-10R1 and IL-10R2 influence receptor rearrangements upon ligand binding. Confocal fluorescent spectroscopy studies would enable the investigation of intracellular receptor rearrangements among the components of the IL-10R complex as well as the Janus kinases Jak1 and Tyk2. This would ultimately give novel insights into the molecular mechanism of the IL-10R complex activation and activation of down-stream Janus kinases Jak1 and Tyk2.

## Supporting information

S1 Fig STAT1 is not required for IL-10 activity. Bone marrow-derived macrophages, dendritic cells and mast cells from wild-type and STAT1^-/-^ mice were tested for their response to IL-10. Cells pre-treated with IL-10 were stimulated with 100 ng/ml LPS and TNF-α expression was determined to asses anti-inflammatory properties of IL-10 in macrophages (A) and dendritic cells (B) (*n* = 3, error bars indicate standard error). Mast cells were cultured for 48 hours in the presence of IL-10, where after cell viability was determined (C) (*n* = 3, error bars indicate standard error).
(TIF)

S2 Fig Flow cytometric analysis of transfected CHO-K1 cells. CHO-K1 cells were analysed by flow cytometry upon co-transfection of IL-10R1 and IL-10R2 constructs. (A) Dual staining for extracellular expression of IL-10R1 and IL-10R2. Pictures are given for the isotype and surface staining upon co-transfection of full IL-10R1 and IL-10R2 constructs and reveals the efficiency of co-transfection. (B) Histograms are given for the intracellular staining of IL-10R2 upon co-transfection of different combinations of IL-10R1 and IL-10R2 constructs.
(TIF)

S3 Fig Flow cytometric analysis of bone marrow-derived cells. Bone marrow-derived macrophages, dendritic cells and mast cells were analysed by flow cytometry for the expression of cellular markers CD11b & F4/80 (macrophage markers), CD11c & MHC-II (dendritic cell markers) or FcεRI & c-kit (mast cell markers). Bone marrow-derived cells from all transgenic mice used in this study show identical phenotypes. Furthermore, macrophages and dendritic cells are distinct cell populations as they have different expression profiles for CD11b, CD11c, F4/80 and MHC-II.
(TIF)
