# Supplementary figures and images for: Re-evaluation of IL-10 signaling reveals novel insights on the contribution of the intracellular domain of the IL-10R2 chain
Source: PLoS One. 2017 Oct 10;12(10):e0186317. doi: 10.1371/journal.pone.0186317 (PMC5634637; doi:10.1371/journal.pone.0186317)

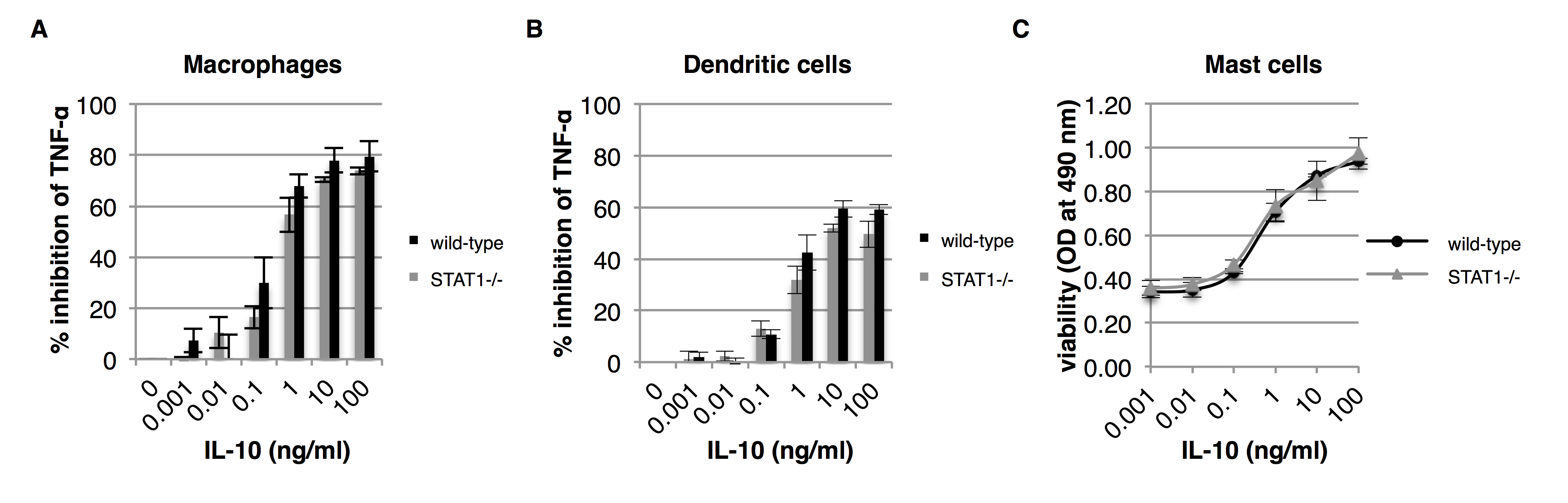

Supplement: S1 Fig — Bone marrow-derived macrophages, dendritic cells and mast cells from wild-type and STAT1-/- mice were tested for their response to IL-10. Cells pre-treated with IL-10 were stimulated with 100 ng/ml LPS and TNF-α expression was determined to asses anti-inflammatory properties of IL-10 in macrophages (A) and dendritic cells (B) (n = 3, error bars indicate standard error). Mast cells were cultured for 48 hours in the presence of IL-10, where after cell viability was determined (C) (n = 3, error bars indicate standard error). (TIF) [file pone.0186317.s001.tif]

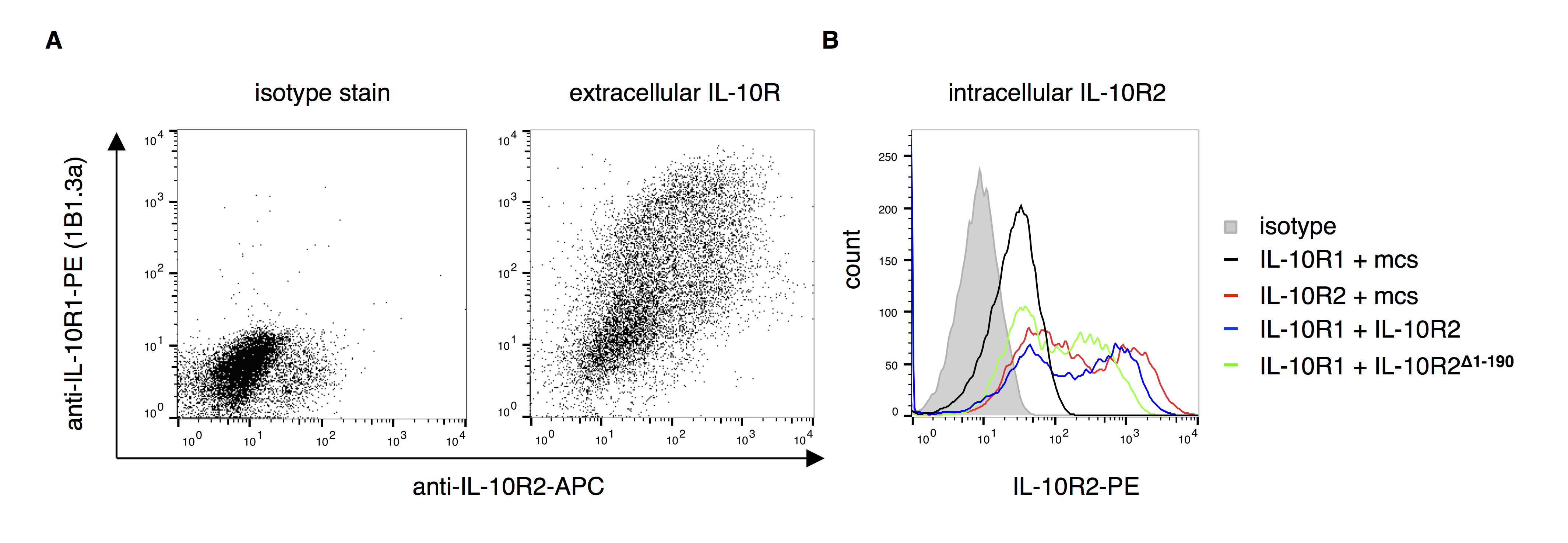

Supplement: S2 Fig — CHO-K1 cells were analysed by flow cytometry upon co-transfection of IL-10R1 and IL-10R2 constructs. (A) Dual staining for extracellular expression of IL-10R1 and IL-10R2. Pictures are given for the isotype and surface staining upon co-transfection of full IL-10R1 and IL-10R2 constructs and reveals the efficiency of co-transfection. (B) Histograms are given for the intracellular staining of IL-10R2 upon co-transfection of different combinations of IL-10R1 and IL-10R2 constructs. (TIF) [file pone.0186317.s002.tif]

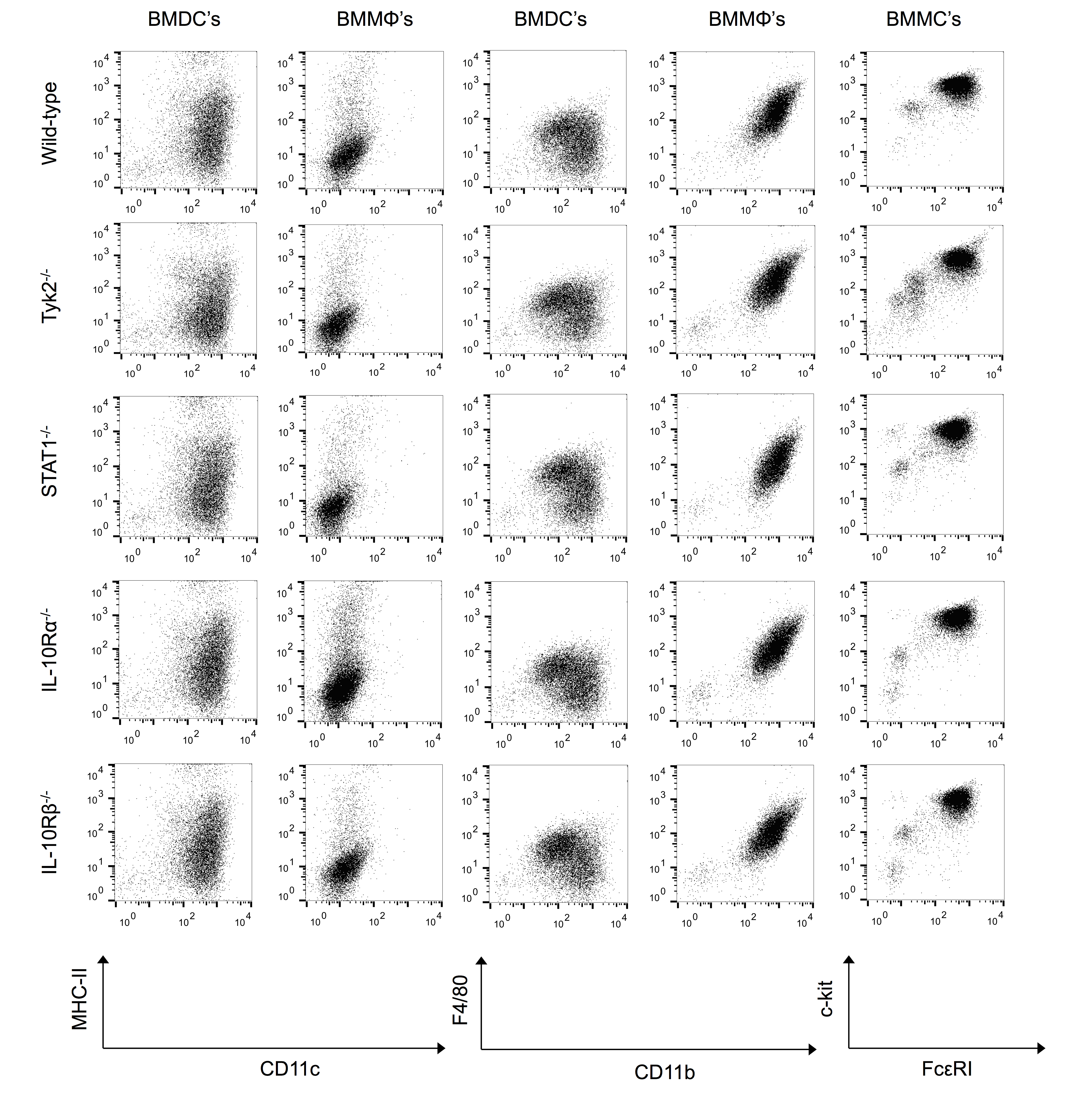

Supplement: S3 Fig — Bone marrow-derived macrophages, dendritic cells and mast cells were analysed by flow cytometry for the expression of cellular markers CD11b & F4/80 (macrophage markers), CD11c & MHC-II (dendritic cell markers) or FcεRI & c-kit (mast cell markers). Bone marrow-derived cells from all transgenic mice used in this study show identical phenotypes. Furthermore, macrophages and dendritic cells are distinct cell populations as they have different expression profiles for CD11b, CD11c, F4/80 and MHC-II. (TIF) [file pone.0186317.s003.tif]
